# Estimation of Coal’s Sorption Parameters Using Artificial Neural Networks

**DOI:** 10.3390/ma13235422

**Published:** 2020-11-28

**Authors:** Marta Skiba, Mariusz Młynarczuk

**Affiliations:** 1 The Strata Mechanics Research Institute of the Polish Academy of Sciences, Reymonta 27, 30-059 Kraków, Poland; 2 Faculty of Geology, Geophysics and Environmental Protection, AGH University of Science and Technology, al. Mickiewicza 30, 30-059 Kraków, Poland; mlynar@agh.edu.pl

**Keywords:** coal properties, artificial neural network (ANN), sorption capacity, effective diffusion coefficient, sorption, methane

## Abstract

This article presents research results into the application of an artificial neural network (ANN) to determine coal’s sorption parameters, such as the maximal sorption capacity and effective diffusion coefficient. Determining these parameters is currently time-consuming, and requires specialized and expensive equipment. The work was conducted with the use of feed-forward back-propagation networks (FNNs); it was aimed at estimating the values of the aforementioned parameters from information obtained through technical and densitometric analyses, as well as knowledge of the petrographic composition of the examined coal samples. Analyses showed significant compatibility between the values of the analyzed sorption parameters obtained with regressive neural models and the values of parameters determined with the gravimetric method using a sorption analyzer (prediction error for the best match was 6.1% and 0.2% for the effective diffusion coefficient and maximal sorption capacity, respectively). The established determination coefficients (0.982, 0.999) and the values of standard deviation ratios (below 0.1 in each case) confirmed very high prediction capacities of the adopted neural models. The research showed the great potential of the proposed method to describe the sorption properties of coal as a material that is a natural sorbent for methane and carbon dioxide.

## 1. Introduction

Coal is a natural sorbent for gases such as carbon dioxide and methane. These gases, present in large amounts in coal mines, are associated with the occurrence of natural hazards. Methane is particularly noteworthy because of its presence in the strata, and its release as a result of mining and geological processes. This hazard is related to the geology of the deposit, i.e., to the type of coal and the presence of cracks or fault zones [1,2,3,4]. The basic tool for evaluating the likelihood of the aforementioned threats in hard-coal seams is analysis of coal’s sorption parameters. In order to determine the properties of a coal–gas system under laboratory conditions, two parameters are primarily used, sorption capacity *a* and effective diffusion coefficient *De*. They complement in situ studies as far as the identification of methane and outburst hazards in mines are concerned. Sorption capacity describes the ability of coal beds to accumulate gas, and the effective diffusion coefficient is decisive for the speed of gas release from coal beds. Among methods used for gas-sorption measurements, gravimetric methods are of importance [5]. In these methods, the amount of sorbed gas is directly determined on the basis of measuring the accumulation of the investigated sorbent’s mass after the sorbate is introduced into the gas system with constant pressure and temperature. These methods have a number of advantages [6,7]; however, due to their time-consuming nature connected to reaching the state of sorption equilibrium by the system, and the high cost of commercially available gravimetric devices, the application of these methods for ongoing forecasting of gas and rock outburst threats is significantly limited. With this in mind, we set out to devise a way to estimate the values of sorption capacity and effective diffusion coefficient, which are seen as the key parameters in the description of coal’s sorption properties, by using artificial neural networks (ANNs).

In recent years, the use of data-analysis algorithms based on artificial-intelligence methods has become increasingly popular. Of these algorithms, ANNs are characterized by high effectiveness. An ANN is a mathematical model of a biological network that mimics the functioning of the human brain in aspects such as pattern classification, forecasting, and decision making based on past experiences. ANNs have a range of advantages that make them highly useful in practical applications. The level of theoretical knowledge required of the user, which is indispensable for effective model construction, is significantly lower than that in the case of traditional statistical methods. Additionally, ANNs are able to generalize obtained knowledge, they are resistant to partial damage in training datasets, and they make it possible to simultaneously process data, which has a positive impact on the speed of action of cooperating neurons. ANNs are increasingly applied in natural, medical, economic, and technical sciences, including mining and geology [8,9,10,11,12]. These networks are also used to predict the mining hazards, including methane hazards, mentioned in current research [13,14,15].

Neural models are applied with substantial success in regression analysis [16,17,18,19], performing the nonlinear transformation of input data in order to approximate output values. Regression issues might be addressed using a feed-forward back-propagation network (FFN), a network with radial basis functions (RBN), or a general-regression neural network (GRNN) [20,21,22].

In this paper, the GRNN model was used to estimate the values of coal’s sorption parameters, such as the effective diffusion coefficient and maximal sorption capacity, on the basis of technical and densitometric analyses, and on the knowledge of the petrographic composition of the coal samples. Results were verified with the use of sorption analyzer IGA-001 (Hiden Isochema, Warrington, UK).

## 2. Materials and Methods

Coal used in the research was obtained from 24 coal beds in the Upper Silesian Coal Basin (Poland). Coal samples were comminuted after being transported to a laboratory. Subsequently, with the dry-sieving method, 4 grain fractions were distinguished for specific purposes. The 10–20 mm grain fraction was used for densitometric analyses, and the 0.50–1.00 mm grain fraction for the microscope petrographic analyses. The 0.125–0.160 mm grain fraction was for sorption measurements, and the grain fraction below 0.20 mm was used in technical analyses [23].

Granular samples (thick polished sections) for petrographic analyses were prepared in line with guidelines specified in ISO norm 7404-2 (methods of preparing coal samples) [24]. Analyses were performed using the AXIOPLAN polarizing microscope (Zeiss, Oberkochen, Germany). The thick polished sections were analyzed in reflected white light, in oil immersion, with 500× magnification. Tests were performed at 1500 evenly distributed measurement points across the surface of the analyzed sample, in line with guidelines specified in ISO norm 7404-3 (method of determining maceral group composition) [25]. Microscopic research was accompanied by measurements of the average reflexivity (i.e., the ability to reflect light) of vitrinite (colotelinite), denoted as R_0_. Measurements were performed in line with the procedure described in ISO norm 7404-5 (method of microscopically determining the reflectance of vitrinite) [26].

The basic characteristics of the investigated coal samples were obtained from results of the technical analyses. The researchers applied the gravimetric method, falling back on procedures specified in ISO norms 562 and 1171 (determination of volatile matter and ash, respectively) for testing solid fuels and hard coal [27,28]. Technical-analysis parameters are expressed as the percentage loss of a sample’s mass in relation to its original mass.

Densitometric analyses were performed with the helium- and quasiliquid-pycnometry methods using analyzers AccuPyc 1340 and GeoPyc 1360, respectively, provided by Micromeritics (Atlanta, GA, USA). Measurement results of the real and apparent density of the coal samples were used as a basis for determining the porosity of these samples.

Parameters that were determined as a result of the above-mentioned analyses constituted a source of basic information on the tested coal samples. Such analyses are routinely performed in coal laboratories and provide information such as the petrographic composition of the samples, moisture and ash content, and internal structure (porosity). These analyses are performed in a relatively short amount of time, and their execution does not require high costs. This is important from the point of view of the methodology proposed in this paper.

Sorption measurements were also conducted, and two parameters were established for each sample: effective diffusion coefficient *D_e_* from the Timofiejew equation [29]:

(1) De=0.308×R2π2×t12,

where

*R*—equivalent radius: $R=\frac{1}{2}\sqrt[{3}]{\frac{2\times d_{1}^{2}\times d_{2}^{2}}{d_{1}+d_{2}}}$;
*d*_1_—minimal grain diameter (lower sieve size, cm),
*d*_2_—maximal grain diameter (upper sieve size, cm),
*t*_1/2_—sorption half-time (s); and

maximal sorption capacity *a_m_* based on sorption isotherm [30]:

(2) $$a=a_{m}\times \frac{b\times p}{1+b\times p},$$

where

*a*—amount of sorbed methane under given equilibrium pressure *p* (m^3^CH_4_/Mg),
*a_m_*—maximal sorption capacity when *p*→∞ (m^3^CH_4_/Mg),
*b*—constant peculiar of coal–methane system (MPa^−1^), and
*p*—free gas pressure (in volume stage, MPa).

Measurements were conducted using the gravimetric method, with the IGA-001 sorption analyzer (Intelligent Gravimetric Analyzer) manufactured by Hiden Isochema (Warrington, UK). The measurements involved tracking changes in sample mass caused by gas sorption/desorption in the function of time [31]. The procedure was performed under constant sorption pressure within the range of 0–1 MPa and under isothermal conditions, in the temperature range from 25 °C (298 K) to 55 °C (328 K) [23].

In our research, the following parameters were used [32,33].

Obtained parameters in the course of technical analysis:

- volatile matter content—V^daf^ (%),
- ash content—A^a^ (%),
- moisture content—W^a^ (%).

Obtained parameters in the course of petrographic analysis:

- vitrinite content—W (%),
- inertinite content—I (%),
- liptinite content—L (%),
- mineral matter content—M (%),
- reflexivity—R_o_ (%),

Parameters obtained in the course of densitometric analysis:

- real density—ρ_r_ (g/cm^3^),
- apparent density—ρ_p_ (g/cm^3^),
- porosity—ε (%).

The research additionally considered the temperature at which the sorption measurements were conducted (T, °C) and the depth of the coal-bed location (H, m). Parameters used in the analyses were normalized in the range [0, 1].

**Figure 1:**
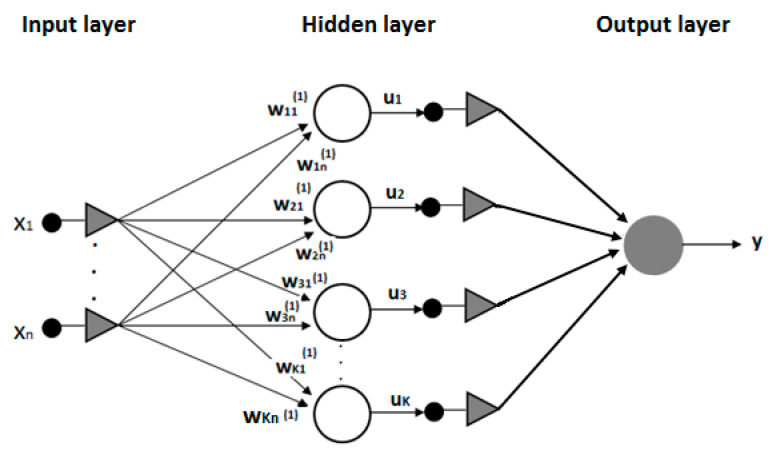
Example of multilayer-perceptron (MLP) network for regression analysis (here, n = 13 and K = 6 or 7 for effective-diffusion-coefficient and maximal-sorption-capacity estimation, respectively).

As a result, each analyzed coal sample was described by means of a 13 dimensional feature vector, which was applied at the input of a multilayer-perceptron (MLP) neural network with unidirectional information flow (Figure 1). That network was used for predicting the values of maximal sorption capacity *a_m_* and effective diffusion coefficient *De*.

**Figure 2:**
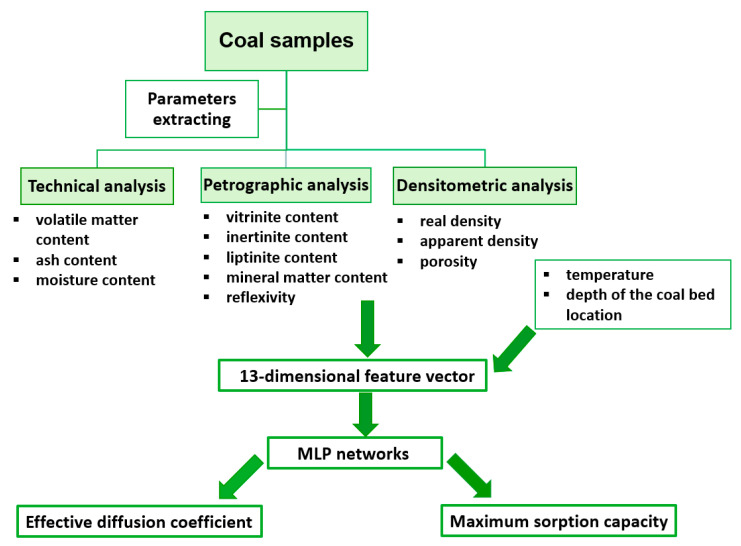
General scheme of performed experiments.

We had 24 coal samples at our disposal for which sorption measurements under 4 temperature levels were conducted: 25, 35, 45, and 55 °C. In this way, we obtained 96 input vectors of the network with the corresponding output values that were subsequently used to train, validate, and test neural models applied in the research. At that point, the time-consuming nature of sorption measurements, determined primarily by the size of the grains of the investigated coal material, should be indicated. In the case of actions involving the 0.125–0.160 mm grain fraction, the time needed to establish a complete sorption isotherm (including necessary time to outgas the sample) at a given temperature value averaged 72 h, which translates into some 10 months of work for all conducted sorption measurements, assuming the measurements would be carried out without pause. The schematic diagram of the performed analyses is presented in Figure 2.

## 3. Prediction Model

Choosing the right number of hidden layers plays an essential role in a neural network solving a given problem [34]. The most common solutions use one or two hidden layers of neurons. On the basis of previous experiences [11,15] and the analysis of relevant scientific sources, we concluded that the optimal neural network for conducting the measurements would be an MLP network with one hidden layer of neurons [35,36]. In this case, it is also important to properly select the number of neurons in the hidden layer and the optimal neuron activation function.

From the available 96 element dataset, 68 elements were randomly selected for the process of training the neural network. The remaining elements were arranged in 2 balanced datasets, validation and test (each consisting of 14 elements). The selection process was repeated 100 times, each time randomly. The validation set was used to evaluate the functioning of the network, and served as a detector of symptoms of the network’s overlearning. The test set was used for the final evaluation of the neural model’s functioning. Analyses were conducted using MATLAB v. 8.5 software (MathWorks, Natick, MA, USA).

At the input of the network, a 13 dimensional feature vector was used: parameters from technical (Parameters 1–3), petrographic (Parameters 4–8), and densitometric (Parameters 9–11) analyses, measurement temperature (Parameter 12), and the depth of the coal-bed location (Parameter 13). The network’s output was constituted by a single linear neuron, which made it possible for the network to reach an unlimited output value range. The tests began with the application of 4 neurons in the hidden layer. That number was established as a geometric mean of the number of inputs and outputs of the network. Thanks to this rule, it was possible to approximately determine the minimal number of neurons in the hidden layer.

In order to determine the optimal size of the hidden layer of the model predicting the first of the considered sorption parameters, i.e., effective diffusion coefficient *De*, analyses were performed for various numbers of neurons in the hidden layer. Analyses also considered the impact of the selected neuron-activation function on the network effects. Two activation functions, widely used in this type of neural network, were subjected to tests: logistic and hyperbolic tangent. In order to train the network, the Levenberg–Marquardt back-propagation algorithm was applied [37]. Results, expressed as average values obtained for 100 randomly selected learning sets, are shown in Table 1.

The adopted matching criterion for the proposed model is expressed as the average percentage prediction error returned by the neural model:

(3) $$C\left(RE\right)=\frac{\sum_{i=1}^{n}\left(\frac{\left|f_{\overset{ˇ}{w}}\left(X_{i}\right)-f_{w}\left(X_{i}\right)\right|}{f_{w}\left(X_{i}\right)}\right)}{n}\times 100\%,$$

where

$f_{\overset{ˇ}{w}}$—prediction value,
$f_{w}$—observed (measured) value,
*X_i_*—test-set element, and
*n*—number of elements in the test set.

Analysis of Table 1 shows that the optimal results for estimating the value of the effective diffusion coefficient (the lowest value of the matching criterion) were obtained using a hidden layer with 6 logistic neurons (MLP 13-6-1).

Corresponding research was conducted for the neural network of predicting the value of maximal sorption capacity *a_m_*. Results, expressed as the average values for 100 randomly selected learning sets, are presented in Table 2.

For estimating the value of the maximal sorption capacity (Table 2), the best results were delivered by a network with 7 hyperbolic tangent neurons in the hidden layer (MLP 13-7-1).

## 4. Results and Discussion

On the basis of analyses described in the previous section, we chose a neural model with a hidden layer of six logistic neurons (MLP 13-6-1) in order to predict the value of the effective diffusion coefficient. The efficiency of the neural model was examined using a test set of 14 examples that were not provided during the neural network’s training process. For 100 random samplings of the learning set, the average prediction error for the parameter values returned by the network, as compared with the actual values determined by using IGA-001 (Formula (1)), was 22.86% (Table 1). The difference between the best match of the neural model and the observed values of the investigated parameter was 6.13%. Values of the effective diffusion coefficient for that match are shown in Table 3.

**Figure 3:**
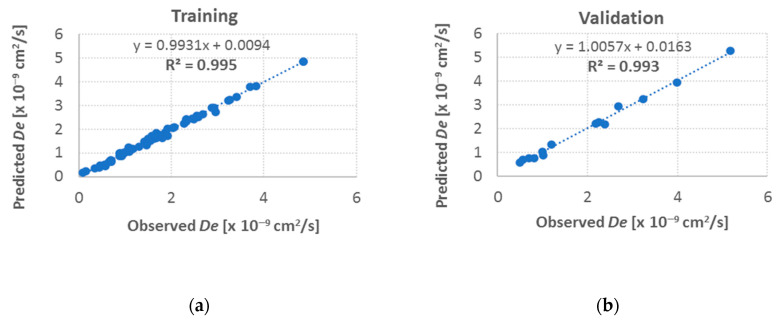

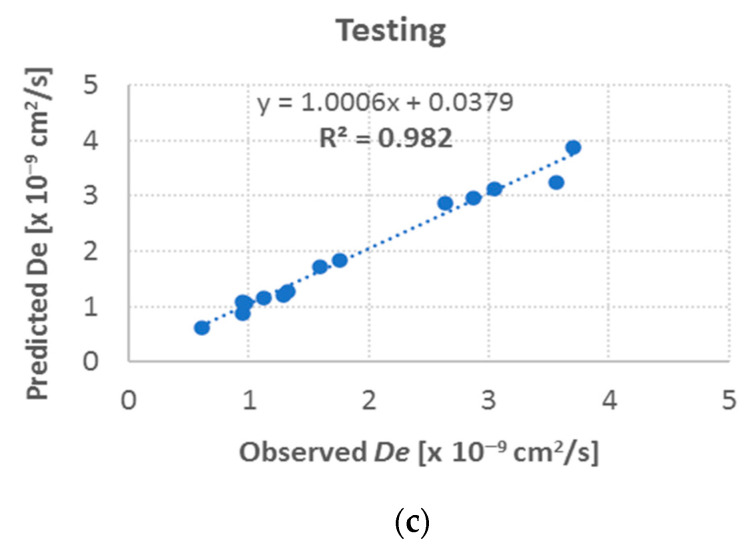
Relationships between effective diffusion coefficient *D_e_* as predicted by neural network and measured values, determined for (**a**) training, (**b**) validation, and (**c**) test sets.

For the best match, correlations between the values provided by the neural network and the observed values were determined. The procedure was performed for the training, validation, and test sets (Figure 3).

As a result of the performed analyses, strong relationships were identified between the theoretical values of the diffusion coefficient and the measured values for the training, validation, and test sets (determination coefficients at 0.98–0.99). Results indicated strong predicting abilities of the investigated neural model. When assessing the regressive model, one should also focus on the value of the standard deviation for training examples and prediction errors. For a very good regression model, the value of the quotient of the standard deviation of prediction errors and the standard deviation of the dependent variable assume values below 0.1. These ratios were independently determined for each of the three datasets, and their values were 0.051, 0.054, and 0.082 for the training, validation, and test sets, respectively. The low values of the determined ratios and the high values of the determination coefficients confirmed very good predicting skills of the neural network described in the research.

In the case of predicting the other discussed parameter, i.e., maximal sorption capacity, the researchers applied a neural model with a hidden layer containing 7 hyperbolic tangent neurons (MLP 13-7-1). For 100 randomly selected learning sets, the average match of parameter values returned by the neural network with the actual values established by IGA-001 (Formula (2)) was 0.89%. The difference between the best match and the observed values of the investigated parameter was 0.22%. Values of maximal sorption capacity for that match are presented in Table 4.

**Figure 4:**
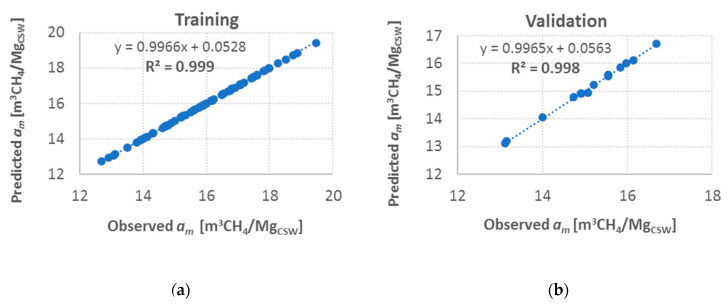

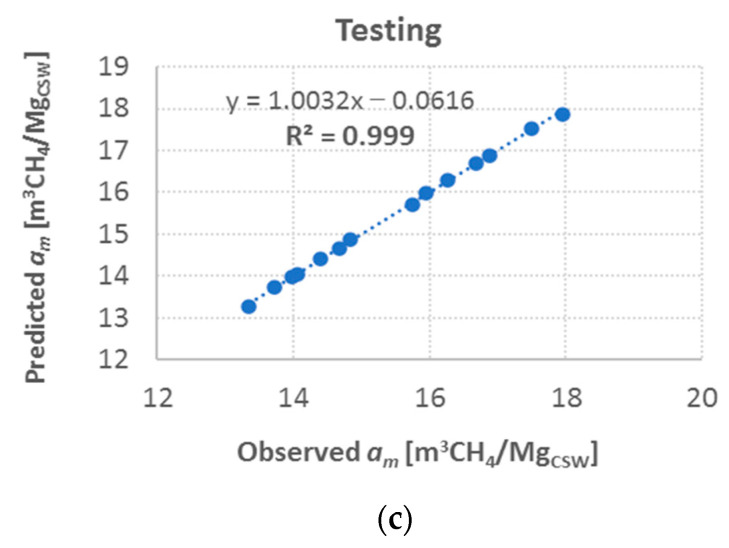
Relationships between maximal sorption capacity *a_m_* as predicted by the neural network and the measured values, determined for (**a**) training, (**b**) validation, and (**c**) test sets.

For the best match, correlations between values returned by the neural network and the observed values were determined. The procedure was performed for the training, validation, and test sets (Figure 4).

As a result of these analyses, strong relationships between the maximal-sorption-capacity values determined with the neural network and the measured values for the training, validation, and test sets were obtained (determination coefficients approximating 1). This indicated the strong predicting abilities of the investigated neural model. Just as in predicting values of the effective diffusion coefficient, standard deviation ratios were determined for the training examples and prediction errors for each of the three datasets in order to evaluate the regression model. The low values of the determined ratios—0.011, 0.032 and 0.017 for the training, validation, and test set, respectively—with the high values of the determination coefficients proved that the applied neural network was characterized by very strong predicting skills.

## 5. Conclusions

This article described research on using artificial neural networks to estimate the maximal sorption capacity and the effective diffusion coefficient of methane in coal samples. In the case of the values of the effective diffusion coefficient, the best estimation results were thanks to an MLP network with six hidden logistic neurons of the logistic activation function. With regard to maximal sorption capacity, the best results were returned by an MLP network with seven hyperbolic tangent neurons in the hidden layer. In each of the investigated cases, theoretical values estimated by the neural network and results obtained using the IGA-001 proved compatible to a substantial extent. With the adopted criterion, in the case of the best match, the relative prediction errors for the analyzed parameters were 6.1% and 0.2% for the effective diffusion coefficient and maximal sorption capacity, respectively. The high determination coefficients (0.982–0.999) obtained in both cases, along with the low values of the standard deviation ratios (below 0.1 in each case) that were determined for the training data and prediction errors, confirmed that the adopted neural models possessed very good prediction skills. This points to substantial practical-application potential for the proposed methodology. Given the time-consuming nature of determining the analyzed parameters with a gravimetric device—conditioned, above all, by the size of grains of the coal material—the proposed methodology could be an effective alternative to the traditional measurement method. It could thus be possible to evaluate gas parameters on the basis of other measurements, which would eliminate the necessity of day-long measurements involving a costly gravimetric device. This would make it possible to use the proposed method to describe the sorption properties of coal as a natural sorbent for methane, which could be used for the ongoing forecasting and evaluation of gas and rock outburst threats in hard-coal mines.

## Figures and Tables

**Table 1:** Selecting neural-network parameters for predicting values of effective diffusion coefficient on the basis of the average prediction error of a neural model.

Hidden-Layer Size	Logistic Activation Function	Hyperbolic Tangent Activation Function
	C (RE, %)	
4	29.41	32.98
5	26.13	30.95
6	22.86	26.32
7	23.37	24.34
8	23.40	25.90
9	25.39	29.43
10	26.25	31.28

**Table 2:** Selecting neural-network parameters for predicting values of maximal sorption capacity on the basis of the average prediction error of a neural model.

Hidden-Layer Size	Logistic Activation Function	Hyperbolic Tangent Activation Function
	C (RE, %)	
4	1.94	1.98
5	1.42	1.41
6	1.34	1.39
7	1.30	0.89
8	1.13	1.09
9	0.97	1.16
10	1.06	1.26

**Table 3:** Effective diffusion coefficient measured using IGA-001 as compared with values returned by the neural network for the best match.

**Observed Value × 10^−9^ (cm^2^/s)**	1.12	3.04	0.95	2.87	0.61	1.32	3.70	3.56	1.29	0.97	2.63	1.76	0.94	1.59
**Predicted Value × 10^−9^ (cm^2^/s)**	1.15	3.12	1.08	2.96	0.62	1.28	3.89	3.24	1.20	1.06	2.88	1.83	0.88	1.73
**Prediction Error (%)**	2.68	2.63	13.68	3.14	1.64	3.03	5.14	8.99	6.98	9.28	9.51	3.98	6.38	8.81

**Table 4:** Maximal sorption capacity measured using IGA-001 as compared with values returned by the neural network for the best match.

**Observed Value (m^3^CH_4_/Mg_CSW_)**	16.89	17.96	14.84	15.74	14.69	13.35	13.99	17.50	15.95	14.07	16.68	16.26	14.41	13.72
**Predicted Value (m^3^CH_4_/Mg_CSW_)**	16.87	17.86	14.88	15.70	14.65	13.28	13.97	17.54	15.99	14.04	16.70	16.29	14.41	13.72
**Prediction Error (%)**	0.12	0.56	0.27	0.25	0.27	0.52	0.14	0.23	0.25	0.21	0.12	0.18	0.00	0.00
